# mTORC1 Mediates Lysine-Induced Satellite Cell Activation to Promote Skeletal Muscle Growth

**DOI:** 10.3390/cells8121549

**Published:** 2019-11-30

**Authors:** Cheng-long Jin, Jin-ling Ye, Jinzeng Yang, Chun-qi Gao, Hui-chao Yan, Hai-chang Li, Xiu-qi Wang

**Affiliations:** 1 College of Animal Science, South China Agricultural University/Guangdong Provincial Key Laboratory of Animal Nutrition Control/National Engineering Research Center for Breeding Swine Industry, Guangzhou 510642, China; jinchenglong1992@163.com (C.-l.J.); cqgao@scau.edu.cn (C.-q.G.); yanhc@scau.edu.cn (H.-c.Y.); 2 Institute of Animal Science, Guangdong Academy of Agricultural Sciences, Guangzhou 510642, China; YEJL2014@163.com; 3 Department of Human Nutrition, Food and Animal Sciences, University of Hawaii, Honolulu, HI 96822, USA; jinzeng@hawaii.edu; 4 Department of Surgery, Davis Heart and Lung Research Institute, The Ohio State University, Columbus, OH 43210, USA; Haichang.Li@osumc.edu

**Keywords:** lysine, mTORC1, satellite cells, proliferation, skeletal muscle growth

## Abstract

As the first limiting amino acid, lysine (Lys) has been thought to promote muscle fiber hypertrophy by increasing protein synthesis. However, the functions of Lys seem far more complex than that. Despite the fact that satellite cells (SCs) play an important role in skeletal muscle growth, the communication between Lys and SCs remains unclear. In this study, we investigated whether SCs participate directly in Lys-induced skeletal muscle growth and whether the mammalian target of rapamycin complex 1 (mTORC1) pathway was activated both in vivo and in vitro to mediate SC functions in response to Lys supplementation. Subsequently, the skeletal muscle growth of piglets was controlled by dietary Lys supplementation. Isobaric tag for relative and absolute quantitation (iTRAQ) analysis showed activated SCs were required for longissimus dorsi muscle growth, and this effect was accompanied by mTORC1 pathway upregulation. Furthermore, SC proliferation was governed by medium Lys concentrations, and the mTORC1 pathway was significantly enhanced in vitro. After verifying that rapamycin inhibits the mTORC1 pathway and suppresses SC proliferation, we conclude that Lys is not only a molecular building block for protein synthesis but also a signal that activates SCs to manipulate muscle growth via the mTORC1 pathway.

## 1. Introduction

Lysine (Lys) is the first limiting essential amino acid for mammals consuming a predominantly cereal-based diet [1,2]. The important role of Lys in promoting skeletal muscle growth has already been demonstrated in animal husbandry, and this effect was attributed to increased protein synthesis [3,4]. Moreover, the functions of Lys in preventing human illnesses, such as osteoporosis and maldevelopment, have been intensively studied to protect human health [5,6]. In contrast, a low Lys diet has been used to treat glutaric aciduria type I and pyridoxine-dependent epilepsy [7,8], despite the fact that Lys deficiency causes severe body growth restriction and a reduction in body weight [9]. Furthermore, to study the mechanism of Lys in governing skeletal muscle growth, it has been reported that the mammalian target of rapamycin complex 1 (mTORC1) pathway is activated by Lys in the skeletal muscle of rats [10]. Additionally, Lys suppresses protein degradation in C2C12 myotubes via greater mTORC1 pathway phosphorylation [11]. However, protein synthesis is controlled by DNA in the nucleus, such that a higher number of cell nuclei in myofibers means greater protein synthesis efficiency [12].

As muscle stem cells are involved in skeletal muscle growth, satellite cells (SCs) are distributed in the basal lamina and sarcolemma of skeletal muscle fibers [13,14]. It has already been established that through proliferation [14], migration [15] and fusion into myotubes to form new nuclei, SCs contribute considerably to muscle fiber hypertrophy [16]. Moreover, in the study of SCs, the mTORC1 pathway is an invaluable index [17,18]. First, mTORC1 is critical for SC participation in skeletal muscle regeneration [19]. Another study showed that mTORC1 is also necessary for RNA-induced mitochondrial restoration in SC activation [17]. Furthermore, the addition of leucine (Leu) could promote proliferation in rat SCs via increasing mammalian target of rapamycin (mTOR) and ribosomal protein S6 kinase 1 (S6K1) phosphorylation [20]. Alway et al. found that a metabolite of Leu, β-hydroxy-β-methylbutyrate (HMB), promotes SC proliferation but does not activate the mTORC1 pathway [21]. Thus, investigating whether Lys could function as a signal regulatory factor that regulates SC proliferation through the mTORC1 pathway to promote skeletal muscle growth is an important endeavor.

In the current work, we aimed to expand our understanding of the role of Lys in governing skeletal muscle growth. Our research was designed to determine the specific skeletal muscle growth of piglets with dietary Lys supplementation in greater detail than a previous study [22]. Importantly, isobaric tag for relative and absolute quantitation (iTRAQ) analysis of the longissimus dorsi muscle displayed differentially expressed proteins related to SCs and the mTORC1 pathway, indicating the potential communication between Lys, the mTORC1 pathway and SCs in skeletal muscle growth. Then, we investigated the changes in proliferation and protein synthesis by accurately controlling Lys supplementation in medium to demonstrate that SC proliferation relies on mTORC1 pathway activation. Moreover, rapamycin was used to confirm the indispensable role of the mTORC1 pathway in the proliferation of SCs with Lys re-supplementation.

## 2. Materials and Methods

### 2.1. Ethics Statement

All animal procedures were performed in accordance with the Guidelines for the Care and Use of Laboratory Animals of South China Agricultural University (Guangzhou, China), and the experiments were approved by the Animal Ethics Committee (SCAU#0158Ethic Committee Approval Number) of South China Agricultural University (Guangzhou, China).

### 2.2. Animals and Sample Collection

The design for the feeding experiment is shown in Table S1. Briefly, a total of 30 Duroc × Landrace × Large White, male, weaned piglets with similar weights were divided randomly into 2 groups from days 0 to 14: the control group was fed a diet containing 1.31% Lys (n = 12), and the Lys deficiency group was fed a diet containing 0.83% Lys (n = 18). On day 15, six piglets closest to the average weight of each group were selected to determine skeletal muscle growth. Then, the remaining piglets in the Lys deficiency group were divided randomly into two groups from days 15 to 28: the Lys deficiency group was fed a diet containing 0.83% Lys (n = 6), and the Lys rescue group was fed a diet containing 1.31% Lys (n = 6). In addition, the remaining piglets in the control group were fed a diet containing 1.31% Lys between days 15 and 28 (n = 6). On day 29, all piglets were slaughtered, and the weight of their skeletal muscle was measured. Longissimus dorsi muscle samples were collected from all of the piglets at days 15 and 29, flash-frozen with liquid nitrogen and stored at − 80 °C.

### 2.3. Amino Acid Detection

To determine the content and concentration of amino acids in longissimus dorsi muscle, samples containing 20 mg protein were weighed and hydrolyzed with 6 mol/L hydrochloric acid (HCL) at 110 °C for 22 h. Then, the hydrolyzed liquid was transferred into a 50 mL volumetric flask with ultrapure water. Then, 1 mL of hydrolyzed liquid was dried by distillation and re-dissolved in 0.02 mol/L HCL. Finally, the amino acid composition was analyzed by an amino acid analyzer (Hitachi L-8900, Tokyo, Japan).

### 2.4. Protein Extraction

Tissue samples (n = 3) were excised and transferred into new tubes containing tissue lysis buffer (1% SDS, 8 mol/L urea) and 1 mmol/L phenylmethanesulfonyl fluoride (PMSF, Sigma-Aldrich, St. Louis, MO, USA). Then, the lysates were homogenized for 4 min using a TissueLyser (CK1000, Thmorgan, Beijing, China) and incubated on ice for 30 min. The lysates were centrifuged at 12,000× *g* and 4 °C for 15 min, and the supernatants were collected. The concentration of proteins was quantified using a micro-bicinchoninic acid assay (BCA) kit (Thermo-Fisher, Waltham, MA, USA) and separated on sodium dodecyl sulfate polyacrylamide gel electrophoresis (SDS-PAGE) gels.

### 2.5. iTRAQ Proteome Analysis

Proteins were treated with tris-(2-carboxyethyl)-phosphine (TECP, Sigma-Aldrich, St. Louis, MO, USA) and iodoacetamide and digested with trypsin. Then, the peptide mixture was labeled using the 8-plex iTRAQ reagent according to the manufacturer’s instructions (Applied Biosystems, Foster City, CA, USA). Because there were eight samples, the peptides were divided into two parts for subsequent detection. For the first peptide group, the control group samples were labeled 115/116, the Lys deficiency group samples were labeled 117, the Lys rescue group samples were labeled 118/119, and the mixture (total of nine samples) was labeled 121. For the second peptide group, the control group samples were labeled 115, the Lys deficiency group samples were labeled 116/118, the Lys rescue group samples were labeled 119, and the mixture (total of nine samples) was labeled 121. Then, equal amounts of peptides from each peptide group were mixed together and vacuum dried.

Then, the peptides were separated by ultra-performance liquid chromatography (UPLC) with a Nano Aquity UPLC system (Waters, Milford, MA, USA) and analyzed in combination with a quadrupole-orbitrap mass spectrometer (Q-Exactive, Thermo-Fisher, Waltham, MA, USA) and an Easy-nLC 1200 (Thermo-Fisher, Waltham, MA, USA) for Nano LC-MS/MS analysis. Finally, the MS/MS data were searched using Protein Discoverer Software 2.1 against the Sus scrofa musculus database (UniProt, https://www.UniProt.org). The false discovery rate (FDR) applied to the control peptide level was defined as lower than 1%. For quantitative analysis, the 0.66 < fold change < 1.5 and *p*-value < 0.05 were the threshold values used to identify the differentially expressed proteins.

All identified proteins were annotated and classified by Gene Ontology (GO, http://www.geneontology.org), and the differentially expressed proteins were then analyzed by GOATOOLS 0.6.5 (https://pypi.org/project/goatools/) for the GO enrichment analysis. Data are available via ProteomeXchange with identifier PXD016396.

### 2.6. Immunohistochemical Analysis

First, the muscle samples were dehydrated with a 20% sucrose solution for 24 h and embedded in Tissue Tek to prepare the cryosections (5 μm, with at least six sections collected from each sample). Then, the tissue slides were incubated with Pax7 (MAB1675, R&D, Minneapolis, MN, USA) and Ki67 (NB500-170, Novus, Miami, FL, USA) at 4 °C overnight. After the slides were washed three times with phosphate-buffered saline (PBS), they were incubated with Alexa Fluor^®^ 488 AffiniPure goat anti-mouse IgG (115-545-003, Jackson, West Grove, PA, USA) and Cy3-AffiniPure Goat anti-rabbit IgG (111-165-045, Jackson, West Grove, PA, USA) at room temperature for 90 min. Next, the slides were washed 3 times with PBS and incubated with 4′,6-diamidino-2-phenylindole (DAPI, Sigma-Aldrich, St. Louis, MO, USA) at room temperature for 5 min. Images were obtained using an immunofluorescence microscope (Ti2-U, Nikon, Tokyo, Japan TYPE, COMPANY, CITY, COUNTRY).

### 2.7. Western Blotting

Protein was extracted from the longissimus dorsi muscle or SCs with lysis buffer (RIPA, BioTeke, Beijing, China) and PMSF (Sigma-Aldrich, St. Louis, MO, USA). Next, the samples were centrifuged at 12,000× *g* and 4 °C for 15 min, and the protein concentration was determined using a micro BCA protein assay kit (Thermo-Fisher, Waltham, MA, USA). A total of 10 µg of protein was separated on 8–10% sodium dodecyl sulfate polyacrylamide gel electrophoresis (SDS-PAGE) gels and then transferred onto polyvinylidene fluoride membranes (PVDF, Millipore, Darmstadt, Germany). After blocking, the membranes were incubated with specific primary and second antibodies (Table S2). Immunoreactivity was detected using an electrochemiluminescence (ECL) Plus chemiluminescence detection kit (Millipore, Darmstadt, Germany) and a Fluor Chem M system (Protein Simple, Santa Clara, CA, USA). The band density was analyzed using ImageJ Analysis Software (https://imagej.nih.gov) after excluding the background density (n = 3). The results were confirmed by three independent experiments with three samples per treatment.

### 2.8. Isolation and Culture of SCs

The method used to isolate, purify and identify the SCs was performed as described previously with modification [23]. In this study, SCs were isolated from the longissimus dorsi muscle of 5-day-old Landrace piglets and cultured in Dulbecco’s modified Eagle’s Medium/Nutrient Mixture F-12 (DMEM/F-12, Thermo-fisher, Waltham, MA, USA) supplemented with 10% fetal bovine serum (FBS, Thermo-fisher, Waltham, MA, USA) at 37 °C and 5% CO_2_. The medium was changed every 48 h.

### 2.9. Lys Depletion and Supplementation

After a 24 h period to allow adhesion, cells were starved for 6 h in FBS- and Lys-free DMEM/F12 medium. Then, the cells were cultured in 500 μmol/L Lys (control) and 0 μmol/L Lys (Lys deficiency) DMEM/F12 medium with 10% FBS for 24, 48 and 72 h to investigate cell proliferation. For proliferation rescue, due to the extreme decrease in proliferation after Lys deficiency for 48 h, we added sufficient Lys for another 72 h at this point. Lys concentrations in DMEM/F12, FBS and culture medium are displayed in Table S3.

### 2.10. Cell Proliferation Assay

For the 3-(4,5-dimethylthiazol-2-yl)-2, 5-diphenyltetrazolium bromide (MTT) assay, 20 μL 5 mg/mL MTT solution (Sigma-Aldrich, St. Louis, MO, USA) was added to each well and incubated for 4 h. Then, the plates were centrifuged at 1400× *g* for 15 min at 25 °C. A total of 150 μL dimethylsulfoxide (DMSO) working solution was added to each well after the supernatants were carefully discarded. The OD value of the product was evaluated using a microplate reader (Bio-Rad, Hercules, CA, USA) at a wavelength of 490 nm (n = 20). For the cell count assay [14,24], SCs were trypsinized and washed with PBS 3 times, and viable cells were counted using a hemocytometer under an automated cell counter (Count Star, Shanghai, China, n = 10).

### 2.11. Flow Cytometry

SCs were seeded at a density of 5 × 10^5^ cells/well in 6-well culture plates (Corning, Corning, NY, USA) to detect the cell cycle distribution. The cultivation process was carried out as described above and according to a method described previously [25]. After harvesting at 24, 48 and 72 h, the cells were fixed in 70% ice-cold ethanol at −20 °C for cell cycle analysis. Before the samples were analyzed by flow cytometry using a Becton Dickinson Fluorescence Activating Cell Sorter Aria (BD Biosciences, San Diego, CA, USA), the cells were centrifuged at 200× *g* and 4 °C for 5 min, re-suspended in 1 mL PBS, treated with 100 μL 200 mg/mL DNase-free RNase, incubated at 37 °C for 30 min, and treated with 100 μL 1 mg/mL propidium iodide (PI, Sigma-Aldrich, St. Louis, MO, USA) at room temperature (25 °C) for 10 min (n = 6).

### 2.12. Protein Synthesis

To measure protein synthesis, a nonradioactive technique called surface sensing of translation (SUnSET) was used [26,27]. In this study, 1 μg/mL puromycin (Millipore, Waltham, MA, USA) was added to all wells for an additional 30 min of culture, and puromycin was detected by western blotting with an anti-puromycin antibody (Millipore, Waltham, MA, USA, Table S2). The total protein concentration was determined by BCA (Thermo-fisher, Waltham, MA, USA).

### 2.13. Immunofluorescence Staining

SCs were cultured for 96 h for the proliferation rescue assay and differentiation rescue assay. First, SCs were fixed in 4% paraformaldehyde for 30 min and then permeabilized with 0.1% Triton-X-100 for 10 min. After blocking with 1% bull serum albumin (BSA) and 10% goat serum for 30 min, the SCs were stained with primary antibodies for 90 min and then probed with goat anti-rabbit IgG (Table S2). In addition, the nuclei were labeled with 4′,6-diamidino-2-phenylindole (DAPI, Sigma-Aldrich, St. Louis, MO, USA) for 5 min at room temperature. Images were obtained using immunofluorescence microscopy.

### 2.14. Rapamycin Inhibition

After Lys deficiency for 48 h, Lys rescue medium was added alone or combined with 20 or 50 nmol/L (nM) rapamycin for another 48 h. After a total of 96 h, cell viability was measured by MTT assay, and cell proliferation was measured by cell count assay. In addition, cell samples were collected to detect protein synthesis and the mTORC1 pathway by western blotting.

### 2.15. Statistical Analysis

The data were analyzed using Statistical Analysis System software (SAS, Version 9.2; SAS Institute, Cary, NC, USA). For control group and Lys deficiency group comparisons, the results were analyzed by t-test. For control group, Lys deficiency group and Lys rescue group comparisons, the mean data were assessed for significance using Tukey’s test. The data are expressed as the mean ± S.E.M. Differences between treatments were considered statistically significant when *p* < 0.05 and extremely significant when *p* < 0.01.

## 3. Results

### 3.1. Skeletal Muscle Growth in Piglets Relies on Dietary Lys Supplementation

To determine the effects of dietary Lys supplementation on the skeletal muscle growth of weaned piglets, we developed the experimental design shown in Supplemental Table 1. After the piglets (initial body weight: control = 8.42 ± 0.11 kg versus Lys deficiency = 8.42 ± 0.08 kg) were fed the Lys-restricted diet for 14 d, we found that the growth of the piglets (final body weight: control = 11.91 ± 0.18 kg versus Lys deficiency = 11.33 ± 0.18 kg) was significantly suppressed (Table S4). In detail, compared with those of the control group, the relative weights of the longissimus dorsi muscle, extensor carpi radialis muscle, semimembranosus muscle, total forequarters muscle and total hindquarters muscle were significantly decreased by dietary Lys deficiency for 14 d (Table S4).

After dietary Lys deficiency for 14 d, the piglets received a diet we supplemented to match the level in the control diet for another 14 d. Obviously, the final weight of the piglets in the Lys rescue group was significantly increased compared with that of the piglets in the Lys deficiency group (Table 1). Moreover, compared with those after Lys deficiency for 28 d, the relative weights of the longissimus dorsi muscle, lateral head of triceps of brachii muscle, extensor carpi radialis muscle, biceps femoris muscle, semimembranosus muscle, semitendinosus muscle, cranial tibial muscle, soleus muscle, lateral head of gastrocnemius muscle and total hindquarters muscle were all increased in the Lys rescue group, which was subjected to dietary Lys deficiency for 14 d and re-supplemented for another 14 d (Table 1). Collectively, these findings suggest that skeletal muscle growth in piglets relies on dietary Lys supplementation.

### 3.2. Lys-induced Skeletal Muscle Growth in Relation to SC Activation Level

In addition to the great change in longissimus dorsi muscle mass with dietary Lys supplementation (Tables S1 and S4), we also found that the Lys concentration in the longissimus dorsi muscle was significantly reduced by dietary Lys deficiency for 14 d (Table S5), whereas it could be rescued by dietary Lys re-supplementation for an additional 14 d (Table 2). Furthermore, the concentrations of threonine (Thr), serine (Ser), glutamate (Glu) and arginine (Arg) showed the same changes as Lys (Tables S2 and S5).

**Figure 1:**
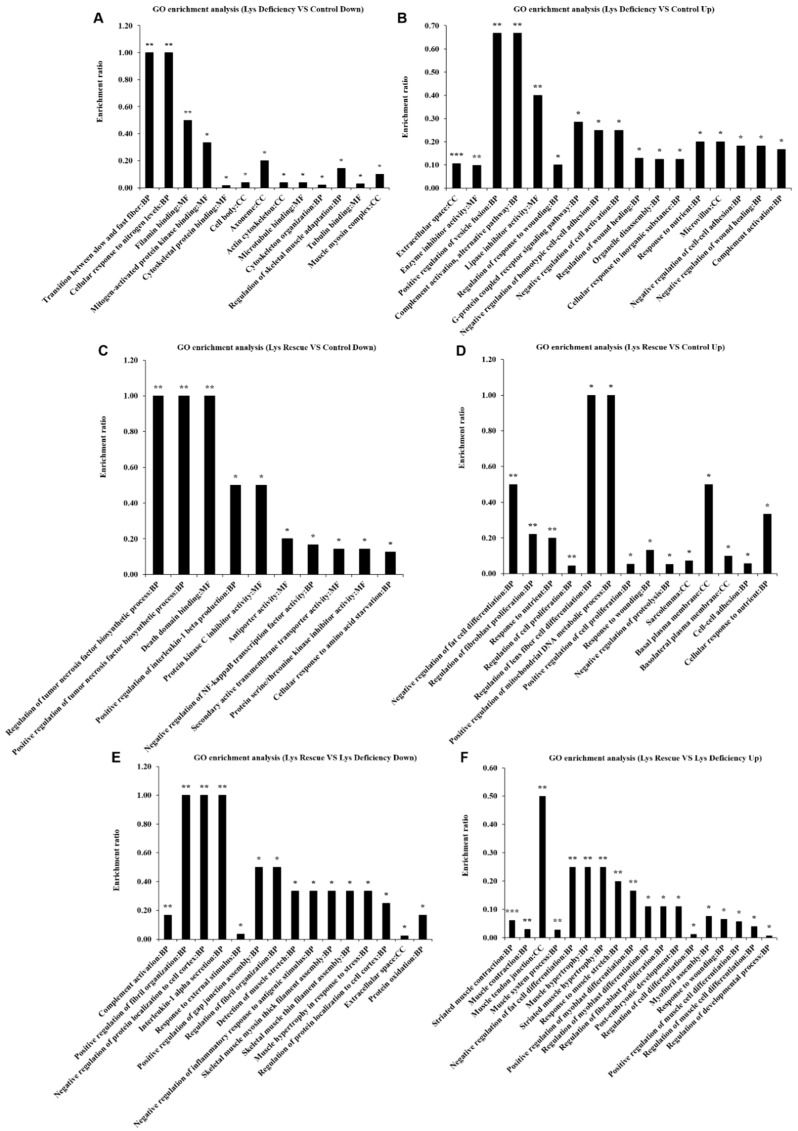
GO enrichment analysis of differentially expressed proteins in the longissimus dorsi muscle on day 28 according to iTRAQ analysis. (**A**) GO enrichment analysis results (Lys deficiency versus control) for downregulated proteins. (**B**) GO enrichment analysis results (Lys deficiency versus control) for upregulated proteins. (**C**) GO enrichment analysis results (Lys rescue versus control) for downregulated proteins. (**D**) GO enrichment analysis results (Lys rescue versus control) for upregulated proteins. (**E**) GO enrichment analysis results (Lys rescue versus Lys deficiency) for downregulated proteins. (**F**) GO enrichment analysis results (Lys rescue versus Lys deficiency) for upregulated proteins. The *x*-axis represents the different GO terms, and the *y*-axis represents the enrichment ratio (the ratio between the protein number enriched in the GO term and the protein number annotated to the GO term; the greater the ratio was, the greater the enrichment found was), * *p* < 0.05, ** *p* < 0.01, *** *p* < 0.001.

Considering these findings, we collected muscle samples for iTRAQ analysis to learn more about the role of Lys in governing skeletal muscle growth (Figure 1). After GO enrichment analysis, we found that proteins related to the transition between slow and fast fibers, filamin binding, mitogen-activated protein kinase binding, cytoskeletal protein binding, actin cytoskeleton, microtubule binding, cytoskeleton organization, tubulin binding and muscle myosin complexes were enriched in the Lys deficiency versus control downregulated proteins (Figure 1A), indicating that the muscle structure was changed.

In addition, in the Lys deficiency versus control upregulated proteins, the enrichment of lipase inhibitor activity, negative regulation of homotypic cell-cell adhesion, negative regulation of cell activation, response to nutrients and negative regulation of wound healing were observed (Figure 1B), and these results suggested that the functions of the muscle cells were disturbed.

Furthermore, proteins related to protein kinase C inhibitor activity, protein Ser/Thr kinase inhibitor activity and cellular response to amino acid starvation were enriched in the Lys rescue versus control downregulated proteins (Figure 1C). Thus, the mTORC1 pathway may play a crucial role in Lys-controlled skeletal muscle growth.

More importantly, the results for the Lys rescue versus control upregulated proteins showed that the proteins related to the negative regulation of fat cell differentiation, regulation of fibroblast proliferation, regulation of lens fiber cell differentiation, positive regulation of cell proliferation, sarcolemma, basal plasma membrane, basolateral plasma membrane and cell-cell adhesion were enriched (Figure 1D). These data suggest that SCs may be activated.

Moreover, the results for the Lys rescue versus Lys deficiency downregulated proteins showed that the positive regulation of fibril organization, regulation of fibril organization and positive regulation of gap junction assembly were enriched (Figure 1E). The results for the Lys rescue versus Lys deficiency upregulated proteins showed that the muscle system process, negative regulation of fat cell differentiation, muscle hypertrophy, positive regulation of myoblast differentiation, regulation of myoblast differentiation, regulation of fibroblast proliferation, regulation of cell differentiation, positive regulation of muscle cell differentiation, regulation of muscle cell differentiation and regulation of developmental process were enriched (Figure 1F). In summary, these results indicate that skeletal muscle growth regulation by dietary Lys supplementation is possibly connected with the mTORC1 pathway and SCs.

### 3.3. SCs and mTORC1 Activity Are Enhanced in Lys-induced Skeletal Muscle Growth

**Figure 2:**
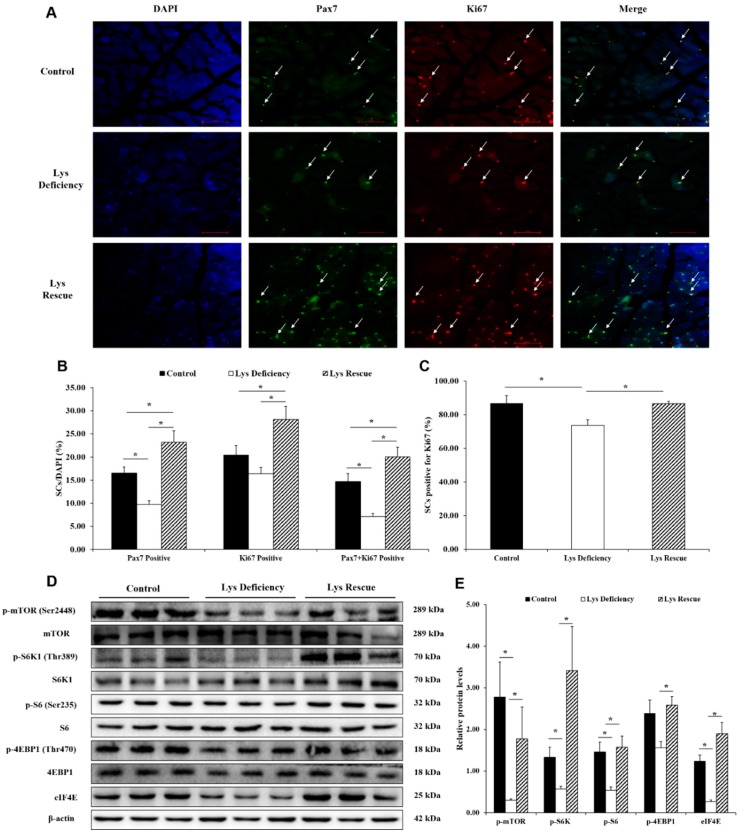
Activation of SCs and protein level of the mTORC1 pathway in the longissimus dorsi muscle on day 28. (**A**) Ki67 (red) and Pax7 (green) staining represents activated SCs during the proliferation period. Bar: 200×. (**B**) Percentage of cells positively stained for Ki67 (red), Pax7 (green) and Ki67 (red) + Pax7 (green) of the total cells (blue, DAPI). (**C**) Percentage of SCs positively stained for Ki67 (red) + Pax7 (green) to Pax7 (green). (**D**) Representative images of key proteins in the mTORC1 pathway detected by western blotting. (**E**) The values represent the ratio of the protein levels of p-mTOR (Ser2448), p-S6K1 (Thr389), p-S6 (Ser235) and p-4EBP1 (Thr470) to the total protein levels and the protein level of eIF4E to that of β-actin, n = 3. The results are shown as the means ± S.E.M. of three independent preparations. Statistical significance was assessed by ANOVA with Tukey’s test, * *p* < 0.05.

The specific marker Pax7 and the proliferation marker Ki67 were detected in SCs in the longissimus dorsi muscle on days 14 and 28 by immunohistochemical analysis (Figures S1A and S2A). Based on the total cells (stained with DAPI), dietary Lys deficiency for 14 d or 28 d significantly decreased the numbers of Pax7-positive cells and Pax7 + Ki67-positive cells compared with the control cells (Figures S1B and S2B). Interestingly, compared with those in the Lys deficiency group, the numbers of Pax7-positive cells, Ki67-positive cells and Pax7 + Ki67-positive cells were all increased in the Lys rescue group and were even higher than those in the control group (Figure 2B). In addition, compared with the control group and Lys rescue group, the Lys deficiency group had a decreased ratio of Pax7 + Ki67-positive cells to Pax7-positive cells, regardless of deficiency for 14 d or 28 d (Figure 1C and Figure 2C). Taken together, these observations suggest that the status of SCs in terms of proliferation in the longissimus dorsi muscle is regulated by dietary Lys supplementation.

In addition, we observed that the key proteins in the mTORC1 pathway, such as p-mTOR (Ser2448), p-S6K1 (Thr389), p-S6 (Ser235), p-4EBP1 (Thr470) and eIF4E (*p* = 0.083), were all inhibited by dietary Lys deficiency for 14 d (Figure 1D,E), and this reduction was observed for samples from the group fed Lys-deficient diets for 28 d (Figure 2D,E). Fortunately, when dietary Lys deficiency was re-established to the control level at 14 d and sustained for another 14 d, the restricted key protein levels of the mTORC1 pathway were all increased (Figure 2D,E). In general, these data indicate that SCs, along with the mTORC1 pathway, are required for Lys-induced skeletal muscle growth.

### 3.4. Cell Proliferation Was Rescued with Increased mTORC1 by Lys Re-supplementation.

**Figure 3:**
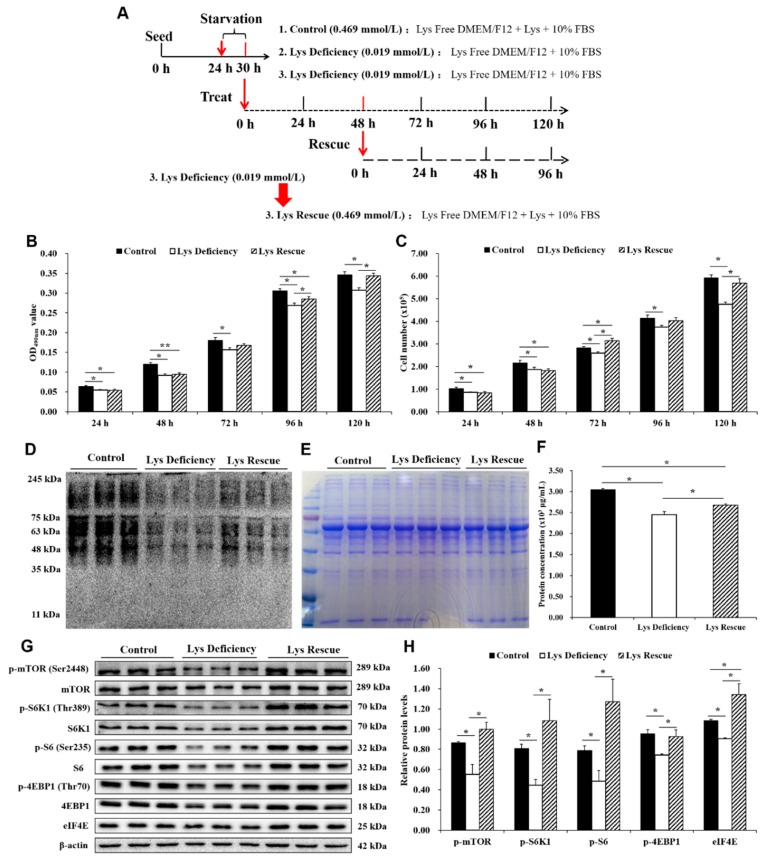
Changes in cell viability, proliferation, protein synthesis and mTORC1 pathway activation after Lys supplementation to sufficient levels. (**A**) Lys supplementation was changed from deficient to sufficient at 48 h, and the cells cultured for another 72 h. (**B**) MTT assays were used to measure cell viability, n = 20. (**C**) Cell proliferation was measured by cell counting assays, n = 10. (**D**) Representative image of the western blotting analyses for puromycin at 96 h, n = 3. (**E**) Coomassie blue staining was used to verify equal protein loading for puromycin measurements at 96 h, n = 3. (**F**) Total protein quantitation using bicinchoninic acid assays at 96 h, n = 3. (**G**) mTORC1 pathway-related proteins were measured by western blotting after Lys supplementation from deficiency for another 48 h (total 96 h). (**H**) The values represent the ratio of the phosphorylated protein levels to the total protein or β-actin level, n = 3. The bars are the means ± S.E.M. from the representative results of three independent experiments. Statistical significance was assessed by ANOVA and Tukey’s test, * *p* < 0.05, ** *p* < 0.01.

To gain further insight into the role of Lys in SCs, Lys supplementation treatment was designed as shown in Figure 3A. In addition, the Lys concentrations in cell culture are shown in Table S3. The MTT assay results showed that cell viability was significantly reduced under Lys deficiency for 48 h (Figures S2A and S3B), whereas cell viability was rescued by Lys re-supplementation for 48 h compared with Lys deficiency for 96 h (Figure 3B). The number of cells detected by an automated cell counter showed that SC proliferation was significantly decreased under Lys restriction conditions for 24 to 120 h (Figures S2B and S3C). In contrast, the number of SCs was increased after Lys was re-supplemented for 24 h after Lys deficiency (Figure 3C). However, cell viability and the number of SCs continued to increase at a slow rate in the deficiency group. This phenomenon might be explained by the existence of Lys in FBS (Figure S3). Because proliferation is determined by mitosis and because cell cycle distribution is a typically evaluated endpoint [25], we further investigated cell cycle distribution using flow cytometry after Lys deficiency for 48 h (Figure 2C–F). Compared with the control conditions, Lys deficiency resulted in an increased percentage of G1 cells at 48 h and 72 h, while the number of cells in the S phase was decreased (Figure 2E,F).

**Figure 4:**
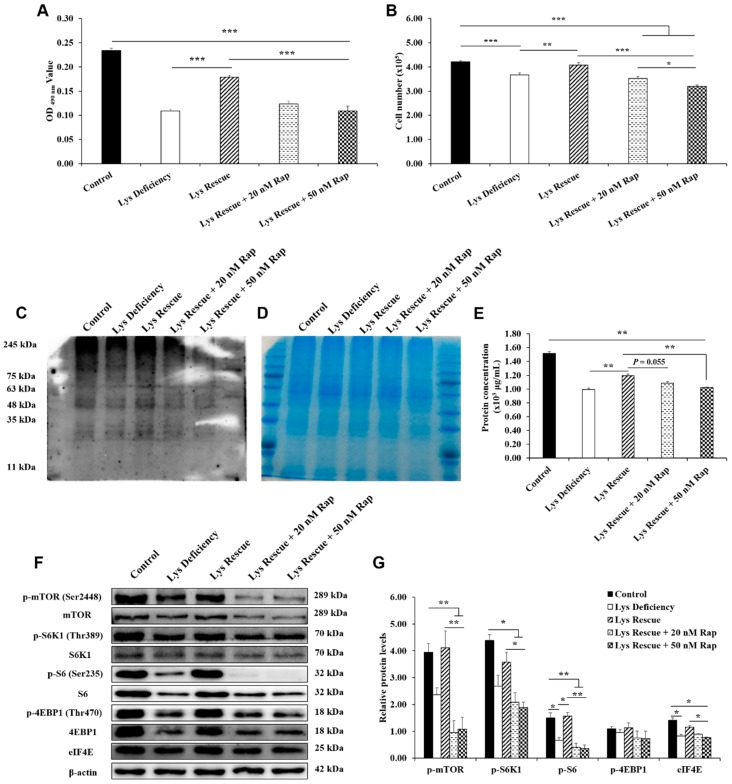
The increased cell viability, proliferation and protein synthesis induced by Lys rescue were inhibited by rapamycin, along with mTORC1 pathway downregulation. After Lys deficiency for 48 h, Lys was added to the medium alone or in combination with 20 or 50 nM rapamycin for another 48 h. (**A**) Cell viability was measured by MTT assays at 96 h, n = 20. (**B**) Cell counting assays were used to measure cell proliferation at 96 h, n = 10. (**C**) SUnSET measurements of protein synthesis were performed by incubating SCs in medium containing puromycin at 96 h. A representative image from the western blotting analyses for puromycin is shown, n = 3. (**D**) Coomassie blue staining was used to verify equal protein loading for puromycin measurements at 96 h, n = 3. (**E**) Total protein quantitation using bicinchoninic acid assays at 96 h is shown, n = 3. (**F**) Western blotting was used to detect the key proteins in the mTORC1 pathway at 96 h. (**G**) The values represent the ratio of the phosphorylated protein levels to the total protein or β-actin level, n = 3. The bars are the means ± S.E.M. from the representative results of three independent experiments. Statistical significance was assessed by ANOVA and Tukey’s test, * *p* < 0.05, ** *p* < 0.01. *** *p* < 0.001.

Moreover, because Lys plays an important role in protein synthesis, the SUnSET assay [26,27] was used to measure changes in protein synthesis in SCs. After Lys deficiency for 48 h, SCs evaluated by puromycin analysis showed an obvious decrease in protein synthesis (Figure S4A). However, protein synthesis was obviously increased after Lys was sufficiently supplemented for another 48 h (Figure 3D). Coomassie blue staining was used to verify equal protein loading (Figure 3E and Figure 4B) [27]. Furthermore, a BCA assay was used to confirm the protein synthesis rate [28], and the results showed that total protein lysis in SCs cultured in Lys-deficient medium was extremely restricted at 48 h (Figure S4C) and was then enhanced after 48 h of Lys supplementation (Figure 3F).

Importantly, to investigate whether Lys-stimulated cell proliferation and protein synthesis were mediated by the mTORC1 pathway, the related proteins were analyzed by western blotting (Figures S4D,E and S3G,H). Compared with the levels in the control group, the levels of phosphorylated mTOR (Ser2448) and its downstream targets, phosphorylated S6K1 (Thr389), phosphorylated ribosomal protein S6 (S6, Ser235) and phosphorylated 4EBP1 (Thr70), and the levels of eukaryotic translation initiation factor 4E (eIF4E) were significantly decreased in the Lys deficiency group at 48 h and 96 h (Supplemental Figure 4E and Figure 3H). However, compared with those under Lys deficiency for 96 h, the phosphorylated protein levels of mTOR and its downstream targets, such as 4EBP1 and S6K1, were restored by Lys supplementation for 48 h after Lys deficiency for 48 h (Figure 3H). Moreover, immunofluorescence staining further demonstrated that p-mTOR (Ser2448) expression was also increased after Lys supplementation (Figure S5). These data indicate that Lys-dependent SC proliferation and protein synthesis are related to mTORC1 pathways.

### 3.5. Lys Rescue of SC Proliferation and mTORC1 Pathway Activation Were Inhibited by Rapamycin

To validate that mTORC1 pathway activity was crucial for Lys-regulated SC functions, SCs were treated with rapamycin under Lys rescue conditions for 48 h. As shown in Figure 4A, we found that the rescued cell viability by Lys re-supplementation was inhibited by the simultaneous addition of rapamycin and reduced to the Lys deficiency level. Moreover, Lys re-supplementation with rapamycin suppressed SC proliferation, and 50 nM rapamycin showed greater inhibition than 20 nM (Figure 4B). Furthermore, the SUnSET assay showed that 20 and 50 nM rapamycin restricted protein synthesis, which was rescued by Lys re-supplementation (Figure 4C). Protein amounts were also verified by Coomassie blue staining, and the results showed equal sample loading (Figure 4D). Apart from that, compared with the control group, all four other groups showed reductions in total protein concentrations (Figure 4E). Importantly, compared with Lys deficiency, Lys re-supplementation increased the total protein concentrations, whereas the increased protein concentrations were decreased by 20 (*p* = 0.055) and 50 nM (*p* < 0.05) rapamycin supplementation (Figure 4E). In addition, compared with control and Lys rescue conditions, Lys re-supplemented with rapamycin significantly decreased the protein levels of phosphorylated mTOR (Ser2448), phosphorylated S6K1 (Thr389), phosphorylated S6 (Ser235) and eIF4E (Figure 4F, G). Although there was no significant difference, the protein level of phosphorylated 4EBP1 (Thr70) was also decreased by 33.18% and 35.17% in the 20 and 50 nM rapamycin groups, respectively, compared to the Lys rescue group. Collectively, these results indicate that Lys-regulated SC functions are mediated by the mTORC1 pathway.

## 4. Discussion

Lys is well known to be one of the most important essential amino acids for body growth [1,3,10,22,29]. However, the mechanisms through which Lys governs muscle mass are still debated. In addition, traditionally recognized muscle mass maintenance has been expanded from protein turnover to cell turnover [12,16]. Thus, the balance between myonuclear accretion and reduction is also an important factor in determining muscle mass, and the function of SC fusion into myotubes is important to study [16,30].

Early studies suggested that changes in whole-body weight were the main responses to dietary Lys supplementation or restriction [3,22,31]. Few studies have focused specifically on skeletal muscle growth. In our study, we found that the growth of almost all separated skeletal muscle was restricted during dietary Lys deficiency, while compensatory growth was shown after Lys supplementation was changed from deficient to sufficient [3,32]. In addition, some weights of specific skeletal muscles were unchanged, which could be caused by differences in myofiber type composition.

In previous studies, skeletal muscle mass accumulation was attributed to the relative efficiency between muscle protein synthesis and degradation [3,4,33]. Nevertheless, our GO enrichment analysis results obtained from the iTRAQ analysis showed that there were great changes in skeletal muscle structure and muscle cell function. Furthermore, SCs were found to participate in Lys-induced skeletal muscle growth. This is consistent with what was mentioned in a previous study, which did not provide compelling evidence [34].

To confirm the implications of the GO enrichment analysis for the longissimus dorsi muscle, we found that the SC proliferation ratios (indicated by Pax7 and Ki67 [35,36,37]) were accurately controlled by dietary Lys supplementation. Consistent with the compensatory growth of skeletal muscle, proliferation also showed the same tendency, especially Pax7 + Ki67-positive SCs. Taking these results together, we believe that SC turnover is required for Lys-induced skeletal muscle growth.

In the case of cell turnover, proliferation is the typical process by which cell numbers are increased by mitosis [14,24]. In this study, the suppressed Lys supply led to reduced cell numbers via changed cell cycle distribution such that there was an increased percentage of G1 phase cells and a decreased percentage of S phase cells. Similarly, a reduction in SC proliferation has also been detected in methionine (Met)- and cysteine (Cys)-restricted cell culture medium [38]. Furthermore, protein synthesis was suppressed directly by Lys deficiency and may thus ultimately cause muscle mass loss [31,32].

In addition to what we found under Lys deficiency conditions, the decrease in cell proliferation was suppressed by changing Lys supplementation from deficiency to sufficiency. These effects may be attributed to the function of Lys as an activator of cell mitotic activity [34]. Furthermore, protein synthesis was rescued by supplementing Lys to Lys-deficient cells. Despite the rescue growth effects of Lys supplementation found in our research, glycine seemed to suppress protein degradation weakly in cells in Lys-deficient medium [11]. These results could explain why Lys seems to play some special functions in life maintenance that have not been previously illustrated, that is, Lys is not used for only protein synthesis [29].

To obtain further insight from our findings, the mTORC1 pathway was measured to investigate the regulatory mechanisms of the Lys relationship with SCs and skeletal muscle growth. Notably, studies have shown that the mTORC1 pathway can be activated by energy, growth factors or nutrients, especially amino acids [38,39]. However, the function of Lys is not as defined as it is for Leu or Arg, as diets supplemented with Leu [40] or Arg [41] promote muscle growth and increase mTORC1 pathway activation. In fact, there has been little research on Lys interactions with mTORC1 in promoting muscle growth. In contrast, the key protein levels in the mTORC1 pathway were not altered after oral Lys administration in rats [33]. More importantly, previous studies showed that the mTORC1 pathway was activated by Lys to suppress protein degradation in vivo and in vitro [10,11]. In the present study, we found that the mTORC1 pathway was inhibited by reducing dietary Lys, and SC proliferation was likewise inhibited in Lys-deficient medium. Furthermore, the related proteins in the mTORC1 pathway were reactivated with complete Lys supplementation. In addition, the indispensable role of the mTORC1 pathway in Lys-governed SC function was verified by rapamycin [20]. We found that the inhibited proliferation and protein synthesis in the Lys deficiency group could be rescued by Lys re-supplementation, whereas these increases were suppressed by the simultaneous addition of rapamycin. Similar to our study, previous studies demonstrated that mTORC1 plays a crucial role in SC function [14,17,18,19]. Therefore, the mTORC1 pathway is necessary for Lys-induced SC activation in vivo and in vitro. However, the molecular mechanisms, such as extracellular Lys sensing in SCs and intracellular mTORC1 activation, need to be further studied.

## 5. Conclusions

**Figure 5:**
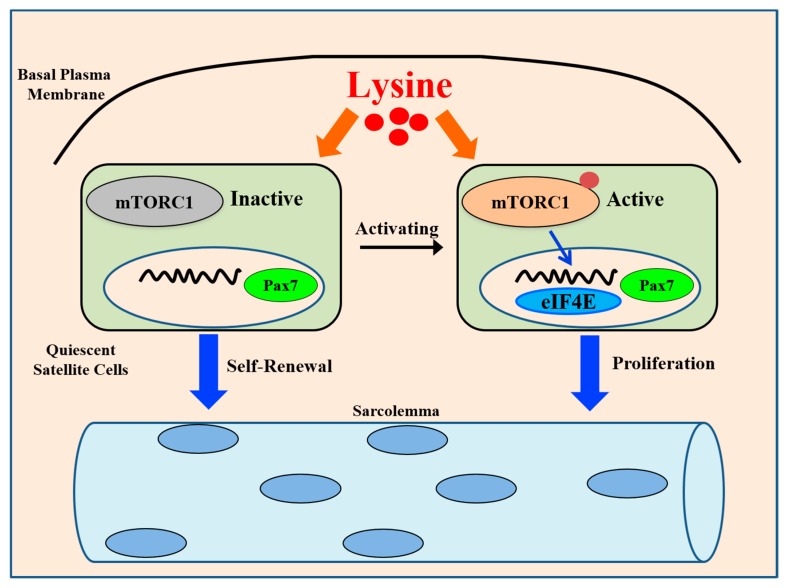
mTORC1 mediates Lys-induced SC activation to promote skeletal muscle growth. Lys supplementation activates the mTORC1 pathway to increase SC proliferation to enhance myogenic potential to promote skeletal muscle growth.

In conclusion (Figure 5), our findings demonstrate that Lys supplementation exerts compensatory growth effects and that the functions of Lys in muscle mass accumulation are mediated by SCs and the mTORC1 pathway. Thus, Lys is not only a molecular building block for protein synthesis but also a signal that activates SCs to regulate muscle growth via the mTORC1 signaling pathway. These findings can provide us with a new target and therapeutic strategy for skeletal muscle regeneration and disease.

## Figures and Tables

**Table 1:** Effect of dietary Lys re-supplementation on the skeletal muscle growth of weaned piglets on 28 d (n = 5, %) ^1^.

Item	Control	Lys Deficiency	Lys Rescue	*p*-Value
Initial Weight (kg)	12.02 ± 0.29	11.32 ± 0.44	11.34 ± 0.35	0.336
Final Weight (kg)	17.95 ± 0.49 ^a^	15.99 ± 0.30 ^b^	17.44 ± 0.46 ^a^	0.018
Longissimus Dorsi Muscle	2.00 ± 0.06 ^ab^	1.86 ± 0.07 ^b^	2.19 ± 0.06 ^a^	0.018
Psoas Major Muscle	0.34 ± 0.01 ^a^	0.28 ± 0.01 ^b^	0.30 ± 0.01 ^b^	0.014
Forequarters muscles				
Infraspinatus Muscle	0.24 ± 0.02	0.22 ± 0.01	0.21 ± 0.01	0.221
Supraspinatus Muscle	0.41 ± 0.02	0.40 ± 0.01	0.42 ± 0.02	0.538
Subclavius Muscle	0.23 ± 0.02	0.25 ± 0.01	0.22 ± 0.01	0.227
Latissimus Dorsi Muscle	0.26 ± 0.03	0.26 ± 0.04	0.27 ± 0.03	0.925
Long Head of Triceps of Brachii Muscle	0.63 ± 0.02	0.63 ± 0.01	0.67 ± 0.02	0.201
Lateral Head of Triceps of Brachii Muscle	0.17 ± 0.01 ^a^	0.14 ± 0.004 ^b^	0.18 ± 0.01 ^a^	0.003
Extensor Carpi Radialis Muscle	0.13 ± 0.003 ^a^	0.12 ± 0.002 ^b^	0.13 ± 0.003 ^a^	0.011
Extensor Muscle of Second- and Third-Digits	0.02 ± 0.001	0.02 ± 0.002	0.02 ± 0.002	0.684
Lateral Digital Extensor Muscle	0.02 ± 0.001	0.02 ± 0.001	0.02 ± 0.002	0.441
Total Forequarters Muscles	2.15 ± 0.07	2.02 ± 0.03	2.15 ± 0.03	0.111
Hindquarters muscles				
Middle Gluteus Medius Muscle	0.54 ± 0.02	0.48 ± 0.02	0.55 ± 0.03	0.102
Superficial Gluteal Muscle	0.17 ± 0.01	0.14 ± 0.01	0.15 ± 0.02	0.457
Biceps Femoris Muscle	1.15 ± 0.01 ^a^	1.04 ± 0.04 ^b^	1.21 ± 0.02 ^a^	0.008
Semimembranosus Muscle	1.36 ± 0.06 ^ab^	1.23 ± 0.05 ^b^	1.46 ± 0.03 ^a^	0.013
Semitendinosus Muscle	0.39 ± 0.02 ^ab^	0.36 ± 0.01 ^b^	0.41 ± 0.01 ^a^	0.043
Tensor fascia Lata Muscle	0.18 ± 0.02	0.17 ± 0.01	0.20 ± 0.01	0.313
Cranial Tibial Muscle	0.04 ± 0.003 ^a^	0.03 ± 0.001 ^b^	0.04 ± 0.002 ^a^	0.045
Long Peroneal Muscle	0.04 ± 0.003 ^a^	0.03 ± 0.001 ^b^	0.03 ± 0.002 ^b^	0.035
Peroneus Tertius Muscle	0.08 ± 0.003	0.08 ± 0.002	0.08 ± 0.002	0.191
Gemelli Muscle	0.27 ± 0.01 ^a^	0.23 ± 0.01 ^b^	0.25 ± 0.01 ^ab^	0.018
Soleus Muscle	0.23 ± 0.005 ^a^	0.19 ± 0.01 ^b^	0.23 ± 0.01 ^a^	0.020
Lateral Head of Gastrocnemius Muscle	0.37 ± 0.005 ^a^	0.31 ± 0.01 ^b^	0.36 ± 0.02 ^a^	0.027
Adductor Muscle	0.18 ± 0.02	0.19 ± 0.01	0.21 ± 0.01	0.301
Total Hindquarters Muscles	4.98 ± 0.12 ^a^	4.49 ± 0.12 ^b^	5.17 ± 0.11 ^a^	0.004

^1^ Values without the same small letters within the same line indicate a significant difference (*p* < 0.05).

**Table 2:** Effect of dietary Lys re-supplementation on the concentrations of amino acids in the longissimus dorsi muscle on day 28 (freeze-dried basis, %) ^1^.

Item	Control	Lys Deficiency	Lys Rescue	*p*-Value
Aspartate	0.11 ± 0.003 ^ab^	0.10 ± 0.006 ^b^	0.12 ± 0.003 ^a^	0.038
Threonine	0.06 ± 0.003 ^a^	0.05 ± 0.003 ^b^	0.06 ± 0.002 ^a^	0.015
Serine	0.05 ± 0.000 ^a^	0.04 ± 0.003 ^b^	0.05 ± 0.002 ^a^	0.002
Glutamate	0.21 ± 0.003 ^a^	0.18 ± 0.012 ^b^	0.21 ± 0.006 ^a^	0.024
Glycine	0.07 ± 0.003	0.06 ± 0.000	0.07 ± 0.006	0.423
Alanine	0.07 ± 0.003	0.07 ± 0.006	0.08 ± 0.003	0.671
Valine	0.06 ± 0.003 ^ab^	0.06 ± 0.003 ^b^	0.07 ± 0.002 ^a^	0.017
Isoleucine	0.07 ± 0.003 ^ab^	0.06 ± 0.003 ^b^	0.07 ± 0.002 ^a^	0.025
Leucine	0.12 ± 0.003 ^ab^	0.11 ± 0.009 ^b^	0.13 ± 0.004 ^a^	0.059
Tyrosine	0.05 ± 0.000	0.05 ± 0.003	0.05 ± 0.003	0.354
Phenylalanine	0.07 ± 0.000 ^ab^	0.06 ± 0.006 ^b^	0.07 ± 0.003 ^a^	0.103
Lysine	0.11 ± 0.003 ^a^	0.09 ± 0.006 ^b^	0.11 ± 0.003 ^a^	0.006
Histidine	0.05 ± 0.003	0.04 ± 0.003	0.05 ± 0.003	0.547
Arginine	0.09 ± 0.000 ^a^	0.08 ± 0.006 ^b^	0.10 ± 0.002 ^a^	0.007

^1^ Values without the same small letters within the same line indicate a significant difference (*p* < 0.05).

## Supplementary Materials

The supplementary materials are available online at https://www.mdpi.com/2073-4409/8/12/1549/s1.
