# Supplementary material for: mTORC1 Mediates Lysine-Induced Satellite Cell Activation to Promote Skeletal Muscle Growth
Source: Cells. 2019 Nov 30;8(12):1549. doi: 10.3390/cells8121549 (PMC6953079; doi:10.3390/cells8121549)
Supplement: Supplementary file 1 [file cells-08-01549-s001.pdf]

Supplementary Materials for

mTORC1 Mediates Lysine-Induced Satellite Cell Activation to Promote Skeletal Muscle Growth

Cheng-long Jin [1], Jin-ling Ye [2], Jinzeng Yang [3], Chun-qi Gao [1], Hui-chao Yan [1], Hai-chang Li [4] and Xiu-qi Wang [1,*]

[1] College of Animal Science, South China Agricultural University/Guangdong Provincial Key Laboratory of Animal Nutrition Control/National Engineering Research Center for Breeding Swine Industry, Guangzhou 510642, China; jinchenglong1992@163.com (C.-l.J.); cqgao@scau.edu.cn (C.-q.G.); yanhc@scau.edu.cn (H.-c.Y.)

[2] Institute of Animal Science, Guangdong Academy of Agricultural Sciences, Guangzhou 510642, China; YEJL2014@163.com

[3] Department of Human Nutrition, Food and Animal Sciences, University of Hawaii, Honolulu, HI 96822, USA; jinzeng@hawaii.edu

[4] Department of Surgery, Davis Heart and Lung Research Institute, The Ohio State University, Columbus OH 43210, USA; Haichang.Li@osumc.edu

* Correspondence: xqwang@scau.edu.cn; Tel./Fax: 86-20-38295462

This PDF file includes:

Fig. S1. Proliferation of SCs and protein levels of the mTORC1 pathway in the *longissimus dorsi* muscle after dietary Lys deficiency for 14 d.

Fig. S2. Lys deficiency suppressed SC viability and proliferation.

Fig. S3. SC viability was decreased by FBS and both Lys deletions.

Fig. S4. Lys deficiency inhibited protein synthesis and downregulated the mTORC1 pathway.

Fig. S5. Immunofluorescence staining for p-mTOR (Ser2448).

Table S1. Feeding experimental design.

Table S2. Antibodies used in the study.

Table S3. Lys concentrations in DMEM/F12, FBS and culture medium.

Table S4. Effect of dietary Lys restriction on skeletal muscle growth in weaned piglets on day 14.

Table S5. Effect of dietary Lys restriction on concentrations of amino acids in the longissimus dorsi muscle on day 14.

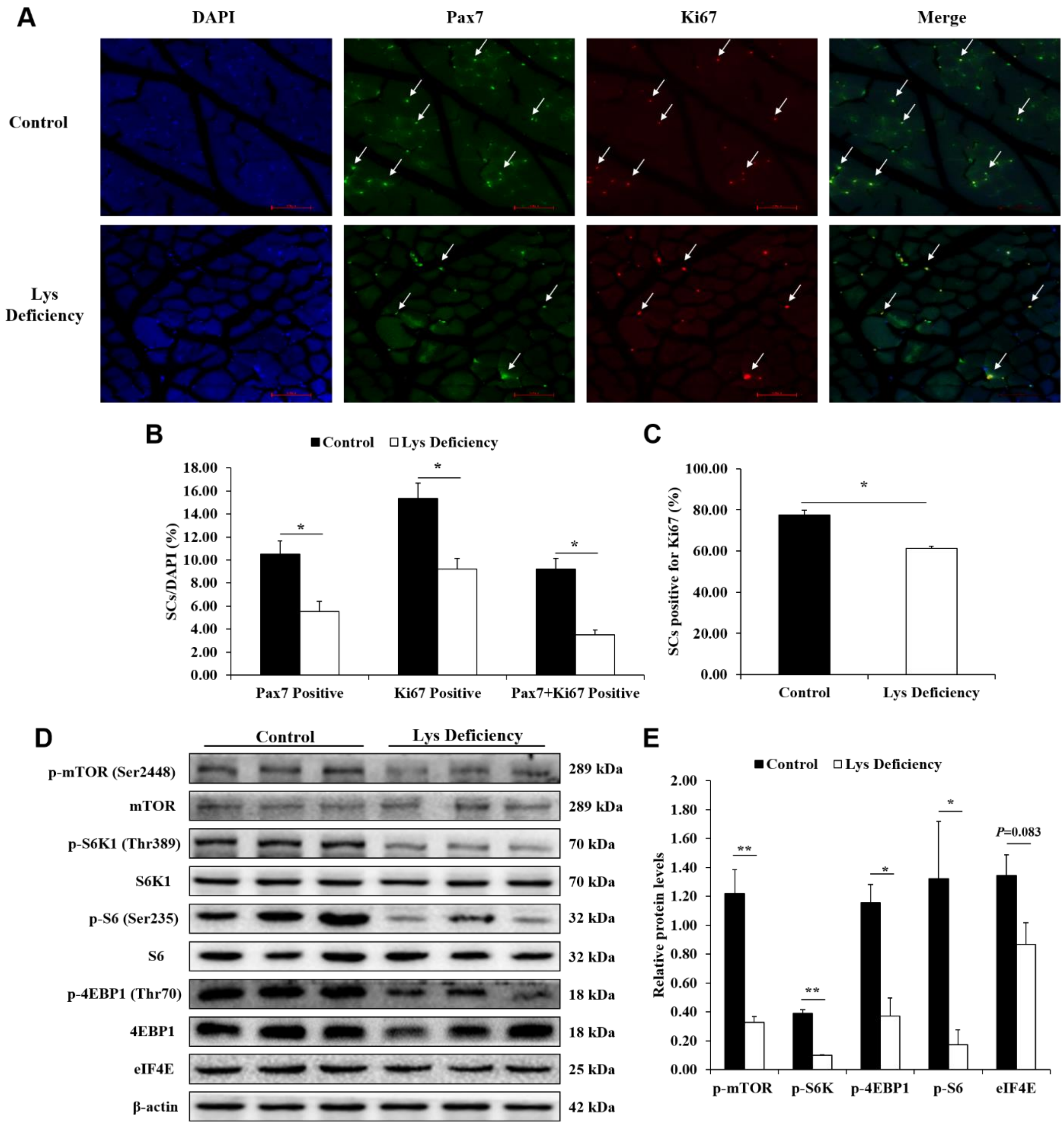

Fig. S1. Proliferation of SCs and protein levels of the mTORC1 pathway in the *longissimus dorsi* muscle after dietary Lys deficiency for 14 d. (A) Ki67 (red) and Pax7 (green) staining represents activated SCs during the proliferation period. Bar: 200×. (B) The percentage of cells positively stained for Ki67 (red), Pax7 (green) and Ki67 (red) + Pax7 (green) to total cells (blue, DAPI). (C) The percentage of SCs positively stained for Ki67 (red) + Pax7 (green) to Pax7 (green). (D) Representative images of key proteins in the mTORC1 pathway detected by western blotting. (E) Values represents the ratio of the phosphorylated protein levels of p-mTOR (Ser2448), p-S6K1 (Thr389), p-S6 (Ser235) and p-4EBP1 (Thr470) to total protein levels and the protein levels of eIF4E to β-actin, n=3. The results are shown as the means ± S.E.M. of three independent preparations. Statistical significance assessed by t-test, * $p < 0.05$, ** $p < 0.01$.

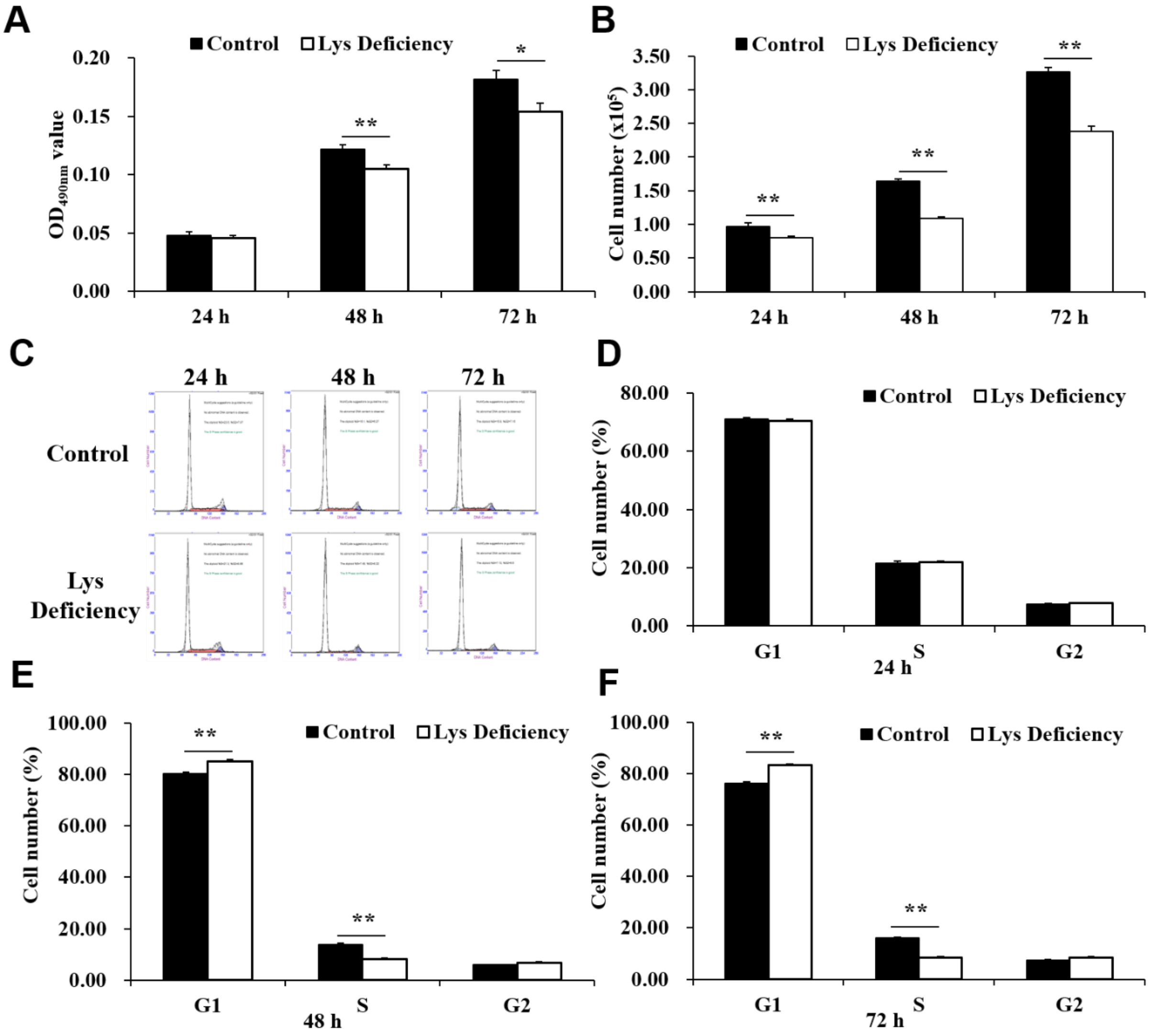

Fig. S2. Lys deficiency suppressed SC viability and proliferation. Cultured SCs was incubated for 24 h in DMEM/F12 medium and cells were starved for 6 h in serum- and Lys-free DMEM/F12 medium. Then cells were cultured in 500 μmol/L Lys (Control) and 0 μmol/L Lys (Lys-deficiency) DMEM/F12 medium with 10 % FBS for 24 h, 48 h and 72 h. (A) MTT (5 mg/mL) was used to measure cell viability through calculating the absorbance value of formazan in viable cells, n=10. (B) Cell proliferation was measured in cell numbers by automated cell counter, n=10. (C-F) The distribution of cell cycle phase was monitored using flow cytometry. Cell cycle distribution statistics were showed at 24, 48, and 72 h after Lys deficiency treatment, respectively, n=6. The results are shown as the means ± S.E.M of three independent preparations. Statistical significance assessed by t-test, * $p < 0.05$, ** $p < 0.01$.

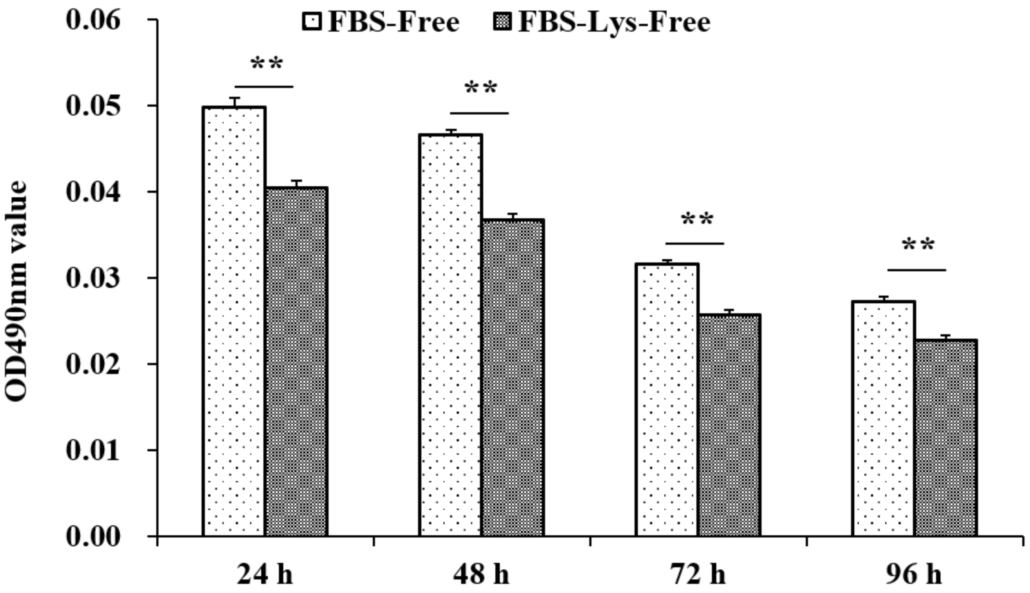

Fig. S3. SC viability was decreased by FBS and both Lys deletions. Cell viability measured via MTT assay. The results are shown as the means ± SEM of three independent preparations. Statistical significance assessed by t-test, ** $p < 0.01$.

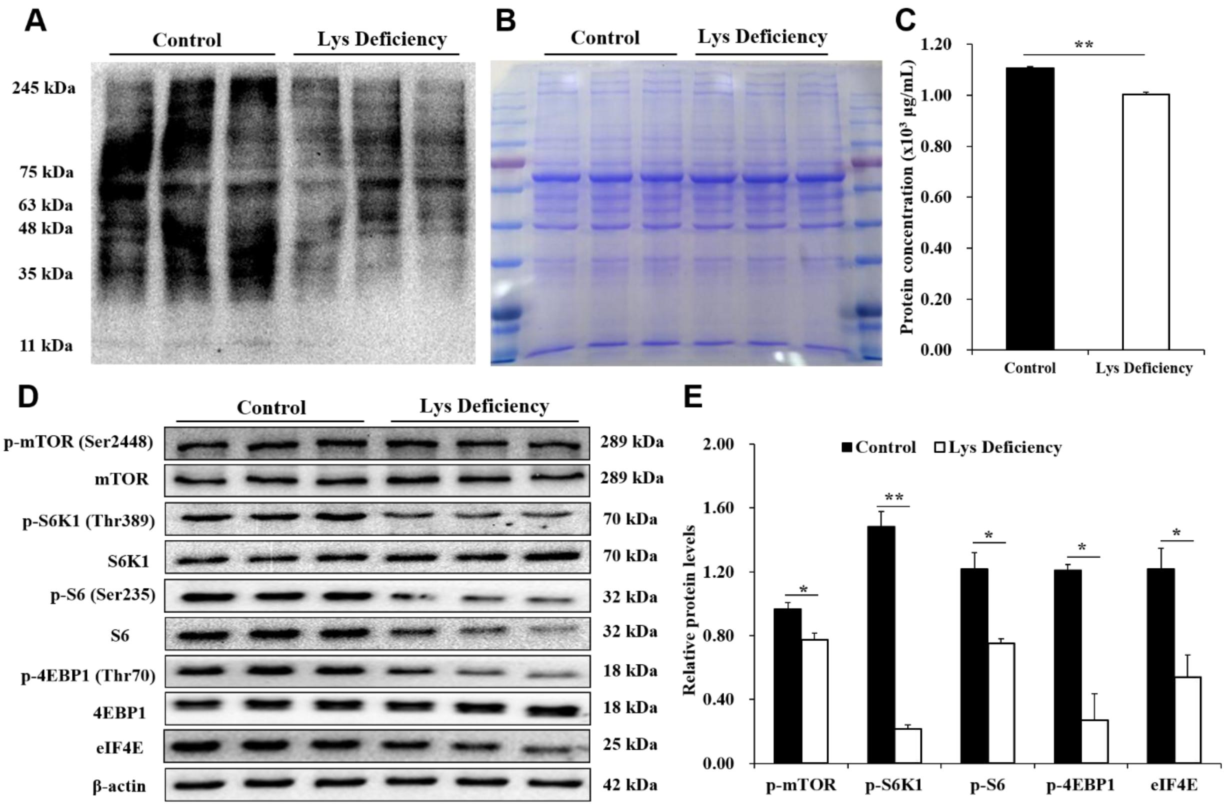

Fig. S4. Lys deficiency inhibited protein synthesis and downregulated the mTORC1 pathway. (A) After 48 h treatment, SUnSET measurements of protein synthesis were performed by incubating SCs in medium containing puromycin. A Representative image from the western blotting analyses for puromycin is shown, n=3. (B) Coomassie Blue staining was used to verify the equal loading of proteins for puromycin measurements, n=3. (C) Total protein quantitation was determined by Micro BCA Protein Assay Kit (Thermo-fisher), n=10. (D-E) Western blotting analysis of key proteins in the mTORC1 pathway after Lys deficiency for 48 h, values are represented as the ratio of phosphorylated protein levels to total protein levels or β-actin, n=3. The results are shown as the means ± S.E.M of three independent preparations. Statistical significance assessed by t-test, * $p < 0.05$, ** $p < 0.01$.

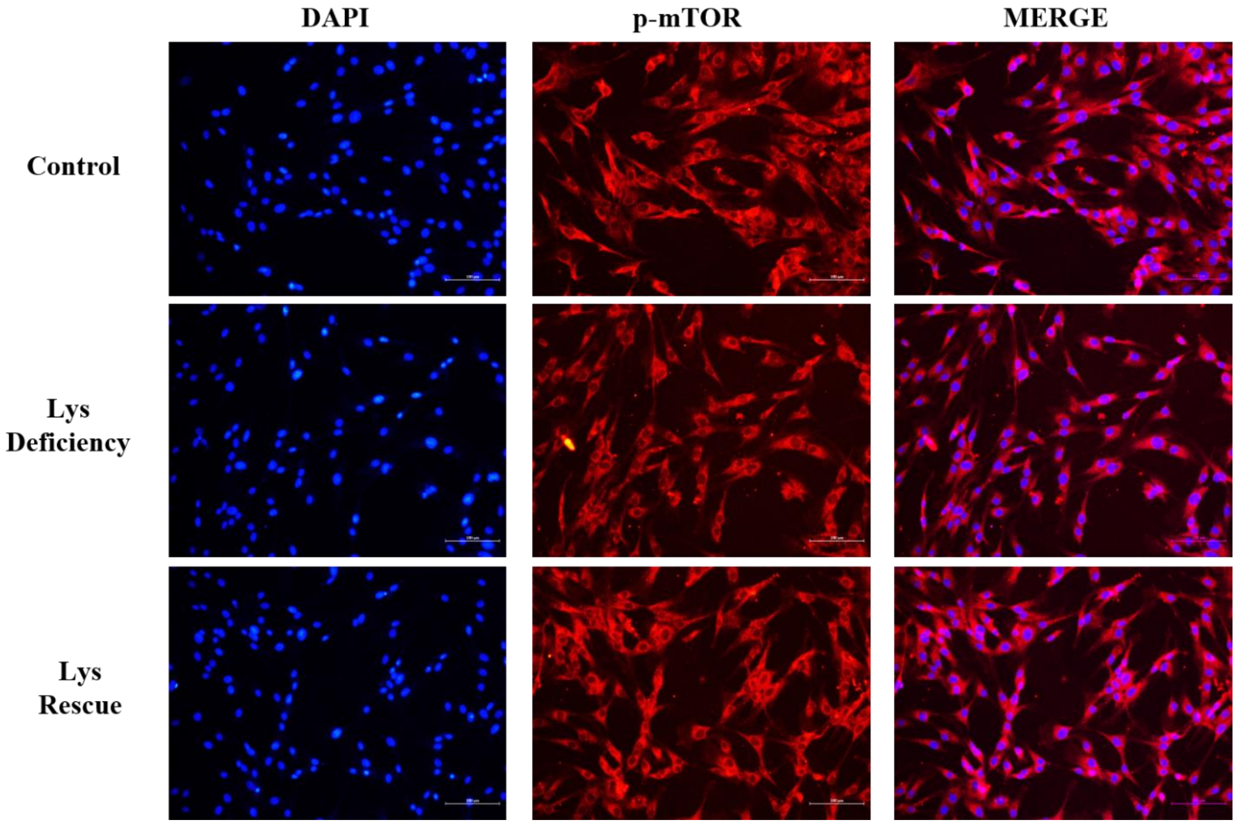

Fig. S5. Immunofluorescence staining for p-mTOR (Ser2448). Immunofluorescence staining was used to examine the changes in p-mTOR (Ser2448) after Lys deficiency for 48 h and Lys supplementation for another 48 h. Bar: 200×.

Table S1. Feeding experimental design.

Item	Control (n=12)	Lys Deficiency (n=18)	
0 – 14 d	Basic diet	Lys restriction diet	
Lys levels:	1.31 %	0.83 %	
**Item**	**Control (n=6)**	**Lys Deficiency (n=6)**	**Lys Rescue (n=6)**
15 – 28 d	Basic diet	Lys restriction diet	Basic diet
Lys levels:	1.31 %	0.83 %	1.31 %

Table S2. Antibodies used in the study.

Antibody	Type	Source	Product Number	phosphorylation site
anti-Puromycin	mouse	Millipore	#MABE343	-
anti-mTOR	rabbit	CST	#2972	-
anti-phospho-mTOR	rabbit	CST	#5536	Ser2448
anti-S6K1	rabbit	CST	#9202	-
anti-phospho-S6K1	rabbit	CST	#9205	Thr389
anti-S6	rabbit	CST	#2217	-
anti-phospho-S6	rabbit	CST	#4858	Ser235/236
anti-4EBP1	rabbit	CST	#9452	-
anti-phospho-4EBP1	rabbit	CST	#9455	Thr70
anti-eIF4E	rabbit	CST	#2067	-
anti-β-actin	rabbit	CST	#4970	-
anti-rabbit IgG	-	Earth	#E030120	-
anti-mouse IgG	-	Earth	#E030110	-

Table S3. Lys concentrations in DMEM/F12, FBS and culture medium.

**Types**	**DMEM/F12 (90%)**	**FBS (10%)**	**Lys Concentration**
Control	0.499 mmol/L	0.194 mmol/L	0.469 mmol/L
Lys Deficiency	0 mmol/L	0.194 mmol/L	0.019 mmol/L
Lys Rescue	0.499 mmol/L	0.194 mmol/L	0.469 mmol/L

Table S4. Effect of dietary Lys restriction on skeletal muscle growth in weaned piglets on day 14 (n=5, %).

**Item**	**Control**	**Lys Deficiency**	***p*-Value**
Initial weight (kg)	8.42±0.11	8.42±0.08	0.978
Final weight (kg)	11.91±0.18	11.33±0.18	0.047
Longissimus dorsi muscle	1.79±0.06	1.59±0.03	0.022
Psoas major muscle	0.29±0.02	0.28±0.04	0.853
**Forequarters muscle**			
Infraspinatus muscle	0.21±0.01	0.19±0.03	0.491
Supraspinatus muscle	0.40±0.02	0.35±0.03	0.200
Subclavius muscle	0.23±0.02	0.22±0.03	0.946
Latissimus dorsi muscle	0.19±0.01	0.15±0.02	0.179
Long head of triceps of brachii muscle	0.65±0.03	0.54±0.05	0.126
Lateral head of triceps of brachii muscle	0.17±0.01	0.17±0.04	0.981
Extensor carpi radialis muscle	0.13±0.01	0.10±0.01	0.028
Extensor muscle of second and third digits	0.02±0.001	0.02±0.003	0.134
Lateral digital extensor muscle	0.02±0.003	0.02±0.003	1.000
Total	2.00±0.06	1.57±0.14	0.022
**Hindquarters muscle**			
Middle gluteus medius muscle	0.50±0.01	0.48±0.02	0.348
Superficial gluteal muscle	0.15±0.01	0.13±0.02	0.302
Biceps femoris muscle	1.06±0.02	0.91±0.07	0.089
Semimembranosus muscle	1.25±0.01	0.99±0.11	0.047
Semitendinosus muscle	0.38±0.01	0.32±0.03	0.099
Tensor fascia lata muscle	0.18±0.01	0.15±0.02	0.211
Cranial tibial muscle	0.03±0.003	0.03±0.004	0.620
Long peroneal muscle	0.04±0.003	0.03±0.003	0.097
Peroneus tertius muscle	0.09±0.003	0.07±0.005	0.067
Gemelli muscle	0.24±0.01	0.23±0.04	0.706
Soleus muscle	0.21±0.01	0.18±0.01	0.060
Lateral head of gastrocnemius muscle	0.33±0.01	0.23±0.06	0.119
Adductor muscle	0.17±0.004	0.19±0.05	0.657
Total	4.61±0.06	4.20±0.12	0.021

$p < 0.05$ indicates a significant difference in the same line.

Table S5. Effect of dietary Lys restriction on concentrations of amino acids in the longissimus dorsi muscle on day 14 (freeze-dry basis, %).

**Amino Acid**	**Control**	**Lys Deficiency**	***p*-Value**
Aspartate	0.12±0.002	0.11±0.004	0.397
Threonine	0.06±0.002	0.04±0.002	0.009
Serine	0.05±0.002	0.04±0.002	0.008
Glutamate	0.21±0.002	0.19±0.006	0.023
Glycine	0.07±0.004	0.06±0.004	0.095
Alanine	0.08±0.002	0.07±0.003	0.172
Valine	0.06±0.002	0.06±0.002	0.094
Isoleucine	0.07±0.002	0.06±0.000	0.070
Leucine	0.13±0.002	0.11±0.004	0.034
Tyrosine	0.05±0.004	0.05±0.002	0.217
Phenylalanine	0.07±0.000	0.06±0.002	0.016
Lysine	0.10±0.002	0.09±0.003	0.045
Histidine	0.05±0.002	0.04±0.002	0.580
Arginine	0.09±0.002	0.08±0.002	0.020

$p < 0.05$ indicates a significant difference in the same line.
